# Thyroid Hormones, Oxidative Stress, and Inflammation

**DOI:** 10.1155/2016/6757154

**Published:** 2016-03-08

**Authors:** Antonio Mancini, Chantal Di Segni, Sebastiano Raimondo, Giulio Olivieri, Andrea Silvestrini, Elisabetta Meucci, Diego Currò

**Affiliations:** ^1^Operative Unit of Endocrinology, Catholic University of the Sacred Heart, 00168 Rome, Italy; ^2^Institute of Biochemistry and Clinical Biochemistry, Catholic University of the Sacred Heart, 00168 Rome, Italy; ^3^Institute of Pharmacology, Catholic University of the Sacred Heart, 00168 Rome, Italy

## Abstract

Inflammation and oxidative stress (OS) are closely related processes, as well exemplified in obesity and cardiovascular diseases. OS is also related to hormonal derangement in a reciprocal way. Among the various hormonal influences that operate on the antioxidant balance, thyroid hormones play particularly important roles, since both hyperthyroidism and hypothyroidism have been shown to be associated with OS in animals and humans. In this context, the nonthyroidal illness syndrome (NTIS) that typically manifests as reduced conversion of thyroxine (T_4_) to triiodothyronine (T_3_) in different acute and chronic systemic conditions is still a debated topic. The pathophysiological mechanisms of this syndrome are reviewed, together with the roles of deiodinases, the enzymes responsible for the conversion of T_4_ to T_3_, in both physiological and pathological situations. The presence of OS indexes in NTIS supports the hypothesis that it represents a condition of hypothyroidism at the tissue level and not only an adaptive mechanism to diseases.

## 1. Introduction

Oxidative stress (OS) is defined as an unbalance between the production of prooxidant substances and antioxidant defenses. The most important prooxidants are the reactive oxygen species (ROS) and reactive nitrogen species (RNS) [1]. The ROS family includes superoxide anion, hydroxyl radical, hydrogen peroxide, and hypochlorous acid. The first three substances are produced* in vivo* mainly by the mitochondrial respiratory chain during the oxidative metabolism of energetic substrates [2, 3]. They are regulators of redox-sensitive pathways involved in cellular homeostasis [4] and influence some transcription factors, in addition to the endogenous antioxidant pool [4–7]. RNS are peroxynitrite, produced by the reaction of nitric oxide (NO) with superoxide, and nitrosoperoxycarbonate, formed by the reaction of peroxynitrite with carbon dioxide. ROS and RNS are considered important pathogenetic factors in different diseases [8]. Among them, a particular pathogenetic role is played by the free radicals, that is, superoxide anion and hydroxyl radical, that are molecules characterized by high chemical reactivity due to a single unpaired electron in the external orbital.

In some cell types, such as leukocytes, endothelial and mesangial cells, fibroblasts, thyrocytes, oocytes, Leydig cells, and adipocytes, ROS generation could play functional roles [9]. Dual oxidases (DUOX), enzymes crucial for hydrogen peroxide generation, are essential for thyroid peroxidase- (TPO-) catalyzed hormone synthesis [10]. Two oxidases of such family are present in thyroid (DUOX1 and DUOX2). They work in conjunction with DUOXA1 and DUOXA2, which are maturation factors that allow DUOX enzymes to translocate to the follicular cell membrane and exert their enzymatic activity [10]. In addition, NADPH oxidase 4 (NOX4) [11] is a new intracellular ROS generating system recently described in the human thyroid gland.

An increased ROS production by the respiratory chain resulting from the rise of the energetic demand or substrate availability [12], as occurs in obesity, or mitochondrial dysfunction or impairment, can produce cell damage and contribute to the pathophysiology of different diseases, such as inflammatory (e.g., rheumatoid arthritis) and cardiovascular (e.g., myocardial infarction) diseases [2]. A pathophysiological role of ROS has been also suggested in diabetes mellitus, in which oxidation accompanies glycation* in vivo* and the antioxidant capacity is decreased, resulting in increased susceptibility to oxidative stress [13].

Different defensive mechanisms that protect against the free radical damage have been characterized in various cellular localizations, including the endoplasmic reticulum, mitochondria, plasma membrane, peroxisomes, and cytosol [2]. Enzymes such as superoxide dismutase (SOD), catalase (CAT) and glutathione peroxidase (GPx), and transition-metal binding proteins, such as transferrin, ferritin, and ceruloplasmin, prevent the production of or rapidly inactivate free radicals. SOD accelerates the dismutation process of superoxide anion in hydrogen peroxide and molecular oxygen that normally occurs with a rate constant 10^4^-fold lower. CAT detoxifies hydrogen peroxide by transforming it in water and molecular oxygen. GPx also participates in hydrogen peroxide detoxification when hydrogen peroxide levels are high. In addition, GPx detoxifies lipid peroxides by transforming them in the corresponding alcohols. “Scavengers” molecules, including both water-soluble, such as albumin, bilirubin, ascorbic acid, urates and thiols, and liposoluble, such as Vitamin E and coenzyme Q_10_ (CoQ_10_), substances interrupt the lipid-peroxidation chain by reacting with and neutralizing the intermediate radicals. The high diffusion rate of scavengers, particularly the liposoluble ones in biological membranes, allows them to intercept radicals and transform them into more stable molecules, thus stopping the radical chain. Sometimes scavengers can be regenerated. A third defensive mechanism uses processes which remove the molecules damaged by the oxidative attack, allowing the reconstitution of normal structures (e.g., specific phospholipases remove the peroxidized fatty acids, making the enzymatic reacylation of damaged molecules possible) [2].

The production of ROS and RNS can occur at the cellular level in response to metabolic overload caused by the overabundance of macronutrients. In addition, mitochondrial dysfunction and endothelial reticulum stress contribute to adipose tissue metabolic derangement in obese patients [14, 15]. ROS generation is further maintained by an inflammatory response, feeding a vicious circle. This picture is worse in pre- and postpubertal children, because puberty alters some inflammatory markers associated with endothelial dysfunction (adipocytokine levels, OS, and insulin sensitivity).

Recent findings suggest that mitochondrial reactive species are signalling molecules that mediate the production of proinflammatory cytokines, thus connecting OS and inflammation. This topic has been extensively studied in cardiovascular diseases [16].

However, besides inflammation, OS can be related to hormonal derangement in a reciprocal way. Some hormones influence antioxidant levels; on the other hand, OS can modify synthesis, activity, and metabolism of hormones. Therefore, OS is related to both systemic inflammation and hormonal derangement. In particular, thyroid hormones play important roles in antioxidant modulation, as demonstrated in different* in vitro* and* in vivo* studies. Reduced glutathione (GSH) is an important cofactor of both antioxidant enzymes and deiodinases, the enzymes responsible for the conversion of thyroxine (T_4_) to triiodothyronine (T_3_). Moreover, plasma levels of small antioxidant molecules, such as Vitamin E and CoQ_10_, and thyroid hormones are closely related to each other [2, 17]. Both hyperthyroidism and hypothyroidism have been shown to be associated with OS and special cases are the autoimmune thyroiditis or the functional picture of low-T_3_ syndrome, observed in acute and chronic nonthyroidal illness syndrome (NTIS) [17–19]. It is still debated whether NTIS represents an adaptive response or a real hypothyroidism at the tissue level. Therefore, studies on OS in NTIS are important to gain knowledge about the pathophysiology of the syndrome itself.

In this review, we firstly examine the relationships between OS and inflammation. Then, we present available data on thyroid hormones and antioxidant regulation. Finally, we report the results of investigations on the relationships between inflammatory mediators and OS in NTIS, in the attempt of hypothesizing a reciprocal influence between tissue hypothyroidism (as primary cause or secondary to inflammation) and OS. Thus, the aim of our review is to discuss and clarify the relationships between thyroid hormones and parameters of OS in the context of the inflammatory diseases.

## 2. Oxidative Stress and Inflammation

Different mediators produced by the adipose tissue may potentially cause an increase of systemic and local ROS and RNS. Thus, the dysregulation of signalling pathway originating in adipocytes, as observed in obese patients, can induce and perpetuate inflammation and OS. Recent studies clearly indicate that the adipose tissue can be considered as an endocrine organ producing different proteins (adipokines) with wide biologic activities. In addition, after maturation from the stage of preadipocytes, the adipocytes gain functions similar to those of macrophages, including the ability to be activated by components of the bacterial wall and to synthesize and secrete cytokines [20]. Moreover, during the periods in which weight gain or loss occurs, the cellular composition of the adipose tissue dynamically changes, showing variations in the levels of various cell types represented in the tissue, in particular vascular and immune cells. The levels of the latter, in particular the macrophages, importantly increase in obese patients. The macrophages seem to play important roles in the pathogenesis of insulin resistance associated with obesity, through the production of Monocyte Chemoattractant Protein-1 (MCP-1) and the modulation of the spreading and the growth of the adipose tissue itself [21]. Monocytes mobilized and attracted by MCP-1, together with neutrophils and lymphocytes T present in the adipose tissue, originate an inflammatory response that is reinforced by the stimulation of the synthesis and secretion of tumor necrosis factor (TNF) by macrophages, in turn induced by the increased production of free fatty acids (FFAs) by adipocytes. In addition, a two-way interaction between adipocytes and macrophages seems to develop, by which the macrophages stimulate the expression and release of MCP-1 from the adipocytes through ROS production. By this way a vicious circle is established, which may promote a chronic inflammatory status gradually more and more intense, typical of obesity and its complications. Finally, the macrophages regulate the remodelling of the adipose tissue when a chronic positive energetic balance ensues. Different pathways are activated in adipocytes depending on whether subtype M1 or M2 macrophages are stimulated, that regulate adipocyte proliferation, growth, and survival. The induced changes are responsible for the appearance of a hypertrophic or hyperplastic obesity. In case of the prevalence the M1 proinflammatory macrophagic subtype, the reduced survival and proliferation of the preadipocytes will cause an inadequate adipocyte reserve; consequently, the energetic backlog, through an excessive hypertrophy, will produce a dysfunctional adipose tissue, which will perpetuate the inflammatory process and, in the long term, produce insulin resistance. Conversely, if the M2 macrophagic subtype is prevalent, the functional pool of preadipocytes will be favoured. They will differentiate into adipocytes, contributing to the formation of an adequate hyperplastic adipose tissue with preserved cell functions and insulin sensitivity [22].

Therefore, obesity is associated with increased secretion of proinflammatory hormones and cytokines (leptin, resistin, TNF-*α*, and interleukin- (IL-) 6) and decreased release of adipokines that downregulate inflammation (adiponectin, IL-10). Recent studies [23] show that not only the amount but also the kind of adipose tissue, as well as the kinds of fats in the diet, influence in different ways this chronic inflammatory state.

Many other mechanisms reviewed by Siti et al. [16] reinforce the link between OS and inflammation. Among these, there is the overexpression of endothelin that induces ROS production in endothelial cells by increasing NADPH oxidase activity [24]; on the other hand, OS causes an increase in angiotensin converting enzyme [25], creating a loop with the previously cited mechanism. Another important mechanism is the OS-induced Ca^2+^ influx, responsible for inflammatory processes [26].

In diabetes, the chronic inflammation, the increase in FFA levels, and the overactivation of the renin-angiotensin system contribute to insulin resistance via OS [27]. TNF-*α*, an important mediator of inflammation, interferes with insulin signals through the activation of the PI3-kinase pathway in endothelial cells [28]. A systemic lipid infusion, that induces acute elevation of plasma FFA levels, causes the activation of the NF-kB pathway, OS, and impairment of endothelium-dependent vasodilatation. In addition, insulin effects on vasodilatation, NO production, and muscle capillary recruitment are blunted by the lipid infusion [29–32]. Regarding this subject, we have shown that a naturally enriched antioxidant diet is capable of improving insulin sensitivity and metformin effects in adult obese patients [33].

Other studies confirmed the link between OS, vascular inflammation, and hypertension-associated vascular changes [34]. Moreover, it is well known that oxidized LDL have a key role in the initiation and progression of the atheromatous plaque [16, 35]; a main role has been recently attributed to the lectin-like oxidized LDL receptor-1 (LOX-1), which is upregulated by the exposure to inflammatory stimuli [36]. The role of the renin-angiotensin system in OS-related injury of endothelial cells has been recently reviewed [37]. Elegant studies conducted in experimental animal models, such as the ApoE knock-out mouse, confirmed an oxidant/antioxidant unbalance in the atherosclerotic process [38–40]. A large number of studies have been published on this topic, which, however, is not among the subjects of the present review. Nevertheless, they overall confirm the association between inflammation and OS.

## 3. The Role of Thyroid Hormones in Antioxidant Regulation

The role of thyroid in the regulation of the antioxidant systems has been recently reviewed in the context of the reproductive endocrinology [41]. It is well known that thyroid function influences the ovarian activity. ROS play physiological roles in the ovary and hypothyroidism, or a low-T_3_ syndrome, can induce ovarian dysfunction by interfering with the antioxidant systems.

OS has been shown to be associated with both hyperthyroidism and hypothyroidism [42]. However, the mechanisms by which OS is generated in these two clinical conditions are different: increased ROS production in hyperthyroidism and low availability of antioxidants in hypothyroidism.

Some complications of hyperthyroidism in target tissues are caused by OS [43]. Thyroid hormones* per se* can act as oxidants and produce DNA damage (contrasted by CAT), probably through the phenolic group, which is similar to that of steroidal estrogens [44]. Many other mechanisms, as previously reviewed [45], can be involved, in particular the enhanced Nitric Oxide Synthase (NOS) gene expression with NO overproduction and the activation of hepatic NF-kB with the consequent increase in cytokines levels which induces ROS production. On the other hand, other mechanisms regulated by thyroid hormones carry out a fine regulation of the oxidative status via autoloop feedback. Among them, we underline the role of Uncoupling Protein- (UCP-) 2 and Uncoupling Protein-3. Data obtained in plants and animals indicate that these molecules have antioxidant activity [46–48]. However, only T_3_ seems to regulate UCP, whereas no effect is exerted by T_4_ [49, 50]. An opposite effect is induced by estrogens, which increase ROS production by repressing UCP [51].

The increased turnover of mitochondrial proteins and mitoptosis also participate in the regulation of the oxidative status, by removing the mitochondria damaged by OS [52]. These processes are regulated by peroxisome proliferator-activated receptor gamma coactivator-1, which in turn is upregulated by T_3_ administration [53].

Thyroid hormones influence lipid composition of rat tissues and consequently the susceptibility to OS. However, the response is tissue-specific, and discrepant effects of T_3_ and T_4_ have been reported. In rat liver, T_3_-induced hyperthyroidism was found to be associated with altered lipid-peroxidation indexes, including elevated levels of thiobarbituric reactive substances (TBARS) and lipid hydroperoxides that are byproducts of lipid peroxidation [45, 53–55]. On the contrary, no changes in TBARS production were found in homogenized livers from rats made hyperthyroid by administration of T_4_ over a 4-week period [56]. No significant changes of TBARS or lipid hydroperoxides were observed in testes of hyperthyroid adult rats as well; however, hyperthyroidism promoted protein oxidation in testes, as indicated by the enhanced content of protein-bound carbonyls [57]. In addition, it should be emphasized that the effects of hyperthyroidism on the activity of antioxidant enzymes, including Mn- or Cu,Zn-SOD, CAT, and GPx, depend on the tissue investigated, with T_3_ and T_4_ having differentiated effects [58].

At the systemic level, hyperthyroidism has been associated with reduced circulating levels of alpha-tocopherol [59, 60] and CoQ_10_ [60, 61] in humans. CoQ_10_ showed a trend toward higher levels in hypothyroidism [61]. Thus, it seems to be a sensitive index of tissue effect induced by thyroid hormones in situations in which drug interference, such as treatment with amiodarone [62], or systemic illness inducing low-T_3_ conditions [63] complicate the interpretation of thyroid hormone levels.

On the other side, data on hypothyroidism and OS in humans are conflicting. In a group of patients with primary hypothyroidism, Baskol et al. [64] found high plasma levels of malondialdehyde (MDA), an OS marker that is formed by lipid peroxidation, and NO, low activity of paraoxonase- (PON-) 1, an enzyme synthetized in the liver with antioxidant properties, and SOD levels not significantly different from those of controls. Interestingly, the treatment with thyroid hormones decreased MDA levels and increased PON-1 activity, even though values similar to those observed in controls were not reached [64]. They hypothesized that in patients with hypothyroidism the prooxidant environment could play a role in the development of atherosclerosis. Elevated MDA levels were also shown in subclinical hypothyroidism [65]. In this setting, the increased OS was attributed primarily not only to the decrease in antioxidants levels, but also to altered lipid metabolism, since a significant correlation among MDA and LDL-cholesterol, total cholesterol, and triglyceride levels was found. Total antioxidant status (TAS) was similar in overt hypothyroidism, subclinical hypothyroidism, and controls.

Excess TSH is known to directly produce OS [66]. Other studies confirmed the lipid peroxidation both in overt hypothyroidism and in subclinical hypothyroidism [67] as indicated by MDA elevation; protein oxidation has been reported as well, with elevation of protein carbonyls [67]. In this study, the correlation analysis suggested that both the TSH increase and the MDA elevation contribute to protein damage. Finally, different studies reported NO elevation [68, 69].

Data on other parameters are more conflicting. As far as PON-1 is concerned, a decreased activity of this enzyme was observed both in hypothyroidism and in hyperthyroidism [70], whereas no significant differences with respect to controls were shown in other studies [68]. Increased levels of TBARS, but also antioxidants, such as SOD, CAT, and Vitamin E, have been also reported [71]. All these parameters correlated with T_3_ and the correlation between T_3_ and CAT remained significant also when corrected for total cholesterol. TBARS elevation was shown in both overt hypothyroidism and subclinical hypothyroidism [69, 72], but these findings were not confirmed in other studies [68, 73].

Another matter of discussion is whether OS is related to hypothyroidism* per se* or to lipid profile alterations caused by thyroid disfunction, as reported above. Indeed, Santi et al. [74] reported OS in subclinical hypothyroidism, as shown by reduced arylesterase and increased TBARS and CAT, but they attributed this pattern to hypercholesterolemia.

We showed low total antioxidant capacity (TAC) levels in hypothyroid patients [75] and increased CoQ_10_ plasma levels in secondary hypothyroidism. This latter finding is mainly to be put in correlation with the metabolic role of CoQ_10_ in the mitochondrial respiratory chain and its consequent reduced cell use in hypothyroid patients. In secondary hypothyroidism, the picture is complicated by concomitant alterations of other pituitary-dependent axes, which can have opposite effect on CoQ_10_ plasma levels. Acromegaly and hypoadrenalism are characterized by low CoQ_10_ plasma concentrations; however, when they are associated with hypothyroidism, this latter has a predominant effect [75, 76].

New perspectives concern DUOX, DUOXA, and NOX4. Cases of hypothyroidism due to mutation of DUOX or DUOXA genes have been reported in the literature [10, 11]. In addition, alterations of NOX4 could be associated with thyroid cancer (via activation by H-Ras oncogene) and Hashimoto's thyroiditis, in which the increased extracellular expression of this enzyme raises Intercellular Adhesion Molecule-1 (ICAM-1) expression and cytokine release [77, 78].

Finally, another study conducted on patients affected by subclinical hypothyroidism secondary to Hashimoto's thyroiditis did not show any difference in endogenous MDA levels between hypothyroid patients and controls; however, MDA induction by the prooxidant 2,2′-azobis-(2-amidinopropane) hydrochloride was markedly augmented in hypothyroid patients. This response in serum was not accompanied by a similar pattern in the LDL fraction: in fact, copper-induced MDA production did not differ in patients affected by subclinical hypothyroidism with respect to controls, whereas it was significantly different from controls in patients with overt hypothyroidism [79]. Studies on patients with thyroiditis should be, however, interpreted with caution, in that both tissue inflammation and systemic inflammation are present in this autoimmune disorder.

The experimental procedures by which hypothyroidism is induced affect the OS findings. Hypothyroidism obtained by surgical thyroid resection in rats was associated with decreased OS in heart [80] and kidney [81]. On the contrary, drug-induced hypothyroidism was associated with increased lipid peroxidation in amygdala [82] and hippocampus in rats [82, 83]. Other cerebral areas, including the cerebellum, remained unaffected [84]. The latter findings, however, were not confirmed in other studies [82, 83]. Similarly, cell damage in various organs, including heart, spleen, liver, lung, and kidney, has been found in animals following methimazole treatment, but not after thyroidectomy [84]. Some studies, however, indicate that the organ damage is not consequent to the hypothyroidism* per se*, but to the drug itself [85, 86].

In the latest years, the attention has been concentrated on the damage induced by OS in certain organs, including liver, bone, skeletal muscle, and particularly the heart [53]. The metabolism of cardiomyocytes depends on serum T_3_, in that these cells lack a significant deiodinase activity [87]. Increased, decreased, or unmodified levels of total SOD, Mn-SOD, Cu,Zn-SOD, GPx, GSH, or Vitamin E have been reported in cardiomyocytes in response to hypothyroidism [88]. Unchanged or decreased levels of various other antioxidant molecules or parameters, such CoQ_9_, CoQ_10_, and TAC, have been also reported. These findings indicate that the evaluation of a single OS parameter is not a reliable index of the cellular oxidative status and the evaluation of TAC depends on the measurement method used.

OS has been also involved in the pathophysiology of schizophrenia. In fact, higher plasma levels of MDA and total plasma peroxides have been found in schizophrenic patients with respect to control subjects, which showed a significant correlation with T_3_ levels [89].

The thyroid itself can be damaged by OS, which occurs in case of iodine excess. This topic has been studied both* in vitro* and in animals fed with a diet rich in iodide [90, 91]. Iodide has a stimulatory action of on hydrogen peroxide generation in thyroid slices and induces thyroid cell apoptosis at high concentrations [92].

Vitamin E has been shown to be protective against the tissue damage induced by peroxyl radicals, mainly not only by preserving the polyunsaturated fatty acids in biological membranes, but also by reducing the activity of NADPH oxidase [53].

## 4. The Model of Low-T_**3**_ Syndrome

Low-T_3_ syndrome is a condition characterized by a reduced peripheral conversion of T_4_ to T_3_ in the presence of normal thyroid hormone secretion. It occurs in a variety of nonthyroidal illness (NTI) and is defined as nonthyroidal illness syndrome (NTIS). The most important acute conditions in which the low-T_3_ syndrome occurs include starvation and eating disorders and critical illness. During starvation (especially carbohydrate deprivation) and nonthyroid illness, deiodination of T_4_ to T_3_ is rapidly inhibited, causing the low-T_3_ syndrome. As the illness progresses to more and more severe stages, a more complex syndrome with low-T_3_ and T_4_ ensues. In critical illness, many other changes of the pituitary-thyroid axis have been shown, including attenuated response to TRH, low tissue uptake of thyroid hormones, and altered thyroid hormone metabolism. A low-T_3_ syndrome caused by the reduced peripheral conversion from the prohormone T_4_ is also observed in different chronic diseases, including chronic kidney disease, liver failure, and chronic inflammatory diseases.

A component of NTIS can be related to cachexia, which is common in chronic systemic inflammation, renal failure, and heart failure. This field has been widely investigated in cancer patients. Cachexia represents a hypermetabolic wasting syndrome with progressive depletion of adipose tissue and skeletal muscle mass, often accompanied by anorexia [93]. Among the mediators of cachexia in cancer patients there are several cytokines and hormones also involved in the pathophysiology of NTIS. They are produced by tumour cells or macrophages surrounding them, as expression of the interaction between the neoplasia and the host environment. The most important are TNF-*α*, IL-1, IL-6, interferon- (IFN-) *γ*, proteolysis-inducing factor (PIF), angiotensin II, and myostatin, a member of the transforming growth factor-*β* superfamily. Interestingly, the signal transduction pathways of many of these substances involve NF-kB, the activity of which is in turn related to ROS levels. In fact, it has been shown that hydrogen peroxide, PIF, and angiotensin II activate NF-kB in myotubes [94] and the treatment of myotubes exposed to TNF-*α*, PIF, or angiotensin II with antioxidants reduces the NF-kB binding to DNA [94, 95]. In addition, it has been reported that the treatment of MAC16 colon-tumour bearing mice with Vitamin E reduces protein degradation in skeletal muscle [95]. Finally, some cytokines, including TNF-*α*, IL-1, IL-6, and IFN-*γ*, mimic leptin signalling, inducing central suppression of appetite [96].

The condition of NTIS is considered as an adaptive response rather than true hypothyroidism. Thyroid replacement therapy is not usually required, but this topic is still debated, since indirect signs of true hypothyroidism at tissue level have been shown. Some molecular mechanisms of NTIS are known, but more studies are necessary to further elucidate its pathogenesis. Indeed, it is probable that a full understanding of the pathophysiological mechanisms at the tissue level will allow the identification of patients who would benefit from replacement therapy. Our discussion will focus on the roles of cytokines and OS in the pathophysiology of NTIS.

The roles of cytokines as key molecules involved in coordinating the hormone, immune, and inflammatory responses to a variety of stressful stimuli are well known [18]. In a series of septic patients studied shortly after admission to the ICU, total T_4_ (tT_4_), free T_4_ (fT_4_), total T_3_ (tT_3_), and TSH plasma concentrations were depressed, and plasma levels of IL-1*β*, sIL-2 receptor, and TNF-*α* were elevated [97], indicating the establishment of central TSH suppression. The hypothalamic-pituitary-adrenal axis was activated as expected. Continuous infusion of IL-1 in rats causes reduction of TSH, free T_3_ (fT_3_), and fT_4_ plasma levels. Higher doses of IL-1 induced a febrile reaction and suppression of food intake, with a cascade of events altering thyroid hormone economy [98]. However, IL-1 did not decrease the hepatic 5′-deiodinase activity that, on the contrary, is typically reduced in NTIS.

TNF is another proinflammatory cytokine that is thought to be involved in many of the alterations associated with NTIS. Infusion of rTNF in man decreases serum T_3_ and TSH and increases reverse-T_3_ (rT_3_) [99]. These findings suggest that TNF could be involved in the IL-6-mediated suppression of the hypothalamic-pituitary axis. However, the involvement of TNF in NTIS pathophysiology was not confirmed in other studies, in which the effects of endotoxin on thyroid hormones in humans were not counteracted by TNF-*α* blockade through specific IgG fusion proteins [100]. TNF-*α* was found in* in vitro* studies to activate NF-kB [101], which in turn inhibits T_3_-induced expression of deiodinase 1 (D1).

An important pathophysiological role in NTIS has been attributed to IL-6, which is often elevated in serum of NTIS patients [102] in an inversely proportional manner with respect to T_3_ levels [103]. Short term infusion of rIL-6 to healthy volunteers [104] suppressed TSH secretion, whereas daily injections over a 6-week period only slightly decreased T_3_ levels and transiently increased rT_3_ and fT_4_ concentrations.

Deiodinases are dimeric selenoproteins that catalyze the stereospecific removal of iodine atoms from the prohormone T_4_, generating the active and inactive isomers of both T_3_ and diiodothyronine (T_2_). Different isoforms are expressed with tissue specificity: D1 and D2, via the deiodination of the outer ring, convert T_4_ to active T_3_; D3, via the inner ring deiodination, converts T_4_ to inactive metabolites: rT_3_ and 3,3′-T_2_ [105, 106]. Phylogenetic analysis suggests that D1 is the oldest vertebrate deiodinase, while D2 is the most recent one; this is in agreement with the key role of D2 as the most specialized and finely regulated member of this enzyme family [106].

Deiodinases play pivotal roles in the regulation of the intracellular levels of active thyroid hormones [107]. D2 is located in the endoplasmic reticulum and plays the primary role in the conversion of T_4_ to T_3_. D1 has lower affinities for the substrates with respect to D2 and seems to be mainly a scavenger enzyme, involved in iodine recycling. Furthermore, the balance between D2 and D3 activities seems to be an important factor in determining the amount of T_3_ available to bind the nuclear receptors. Different mechanisms regulate the expression of deiodinase genes (DIO1, DIO2, and DIO3), first of all the levels of thyroid hormones: hyperthyroidism suppresses D2 activity and DIO2 expression, whereas hypothyroidism exerts the opposite effects [108]. The ubiquitination of the enzymes, which can be reversible to assure the appropriate protein homeostasis, is a mechanism of finer regulation of deiodinase activity [109].

D2 plays important roles in the regulation of the energetic balance as well. It has been shown that animal exposure to low temperatures activates D2 in brown adipose tissue through catecholamine-induced cAMP production. The resulting increase in T_3_ levels induces thermogenic genes, including UCP-1 [110]. In addition, DIO2 expression is upregulated by bile acids in the brown adipose tissue of mice through the increase in cAMP levels. When fed with a high fat diet supplemented with bile acids, the animals do not gain weight, showing a resistance to diet-induced obesity, and this effect is absent in D2 knock-out animals [111, 112].

Recent studies on the effects of IL-6 on both endogenous cofactor-mediated and dithiothreitol-stimulated deiodinase activity in human cell lines [112] have shown that T_3_ generation by D1 and D2 is suppressed by IL-6, despite an increase in expression of deiodinases. The inhibitory action of IL-6 is prevented by the addition of N-acetyl-cysteine (NAC), an antioxidant that restores intracellular GSH concentrations, suggesting the involvement of prooxidant substances in IL-6-induced effects.

Finally, the interaction between the complex network of cytokines and the hypothalamic-pituitary-thyroid axis probably plays pathogenetic roles in NTIS, even though it is not possible to build a simplistic model [18]. Also the role of cytokines in eating disorders and related thyroid hormone alterations has been recently reviewed [113].

Different conditions in which NTIS develops are associated with OS, due to augmented production ROS or RNS [114]. Since thyroid hormones, as above discussed, increase ROS generation, low-T_3_ could be viewed as a compensatory mechanism. In fact, low-T_3_ concentrations would be associated with decreased metabolic rate that would reduce further radical generation. Cytosolic thiols, particularly GSH, and Thioredoxin (Trx), which are also deiodinase cofactors, contribute to the maintaining of a reducing intracellular environment. Thus, their depletion, consequent to their buffering effect on radical propagation, could interfere with the conversion of T_4_ to T_3_ [115]. The nuclear sequestration of SECIS binding protein 2 (SBP2), which reduces the incorporation of selenocysteine residues in the selenoproteins [116], might be another mechanism. It is well known that IL-6 induces OS, so that a unifying mechanism might be that cytokine-induced OS alters secondarily the expression and activity of deiodinases [115]. The contribution of the reduction in the levels of thiol cofactor of deiodinases, consequent to the increase in intracellular ROS concentrations, has been suggested by other authors [117].

On the basis of the pathophysiological studies available in the literature, we can conclude that the alterations of the pituitary-thyroid axis depend not only on the severity of the disease, but also on the inflammatory response and the patients' nutritional status. They also indicate that low-T_3_ is simply not an adaptive mechanism, but it is associated with tissue hypothyroidism and OS.

A special, reevaluated role could be played by selenium. This essential trace element exerts complex effects on the endocrine system, due to its antioxidant capacity; it is a cofactor of GPx and Trx reductase (TrxR), enzymes that protect the cells from the oxidative damage [118]. On the other hand, selenium is involved in the mechanisms of deiodination: a proposed model involves the formation of selenenyl iodide intermediate [119], even though the catalytic mechanisms and the regulation of deiodinases by selenium are not fully understood [120]. Thus, because of its double function, molecules that compete with this element could, in a reciprocal way, connect hypothyroidism due to low-T_3_ and OS. This hypothesis is supported by the evidence that NAC, an antioxidant that restores intracellular GSH levels, prevents the IL-6-induced effects on the intracellular redox state [121, 122]. In addition, the administration of sodium selenite in cells expressing deiodinases decreases the IL-6-induced ROS production and carbonyl protein content and enhances GPx and TrxR activities [123].

Also deiodinases may be involved in NTIS pathophysiology, with possible tissue specificity [124]. DIO1 is a T_3_-responsive gene; thus, D1 activity and intracellular T_3_ concentrations can affect each other in a reciprocal way. D1 activity has been shown to be suppressed in hepatocytes. The activity of D2 has been reported to be reduced [125], unchanged [126], or increased [127] in skeletal muscle. An increase in DIO2 expression in skeletal muscle has been reported in mice during chronic inflammation that has been linked to enhanced CREB signaling [128]. On the contrary, skeletal muscle DIO2 expression was found to be decreased in sepsis and this decrease was related to the reduction in food intake [129]. DIO2 expression increases in lung and in endothelial cells following LPS-induced injury [130] and in hepatic resident macrophages during acute and chronic inflammation [128]. As far as D3 is concerned, a decrease in DIO3 mRNA levels has been reported in liver during inflammation and sepsis [131, 132]. On the contrary, hepatic expression and activity of D3 were found to be increased in rabbits with prolonged critical illness [133]. Similarly, D3 activity was found to be increased in the skeletal muscle of critically ill patients [134] and in patients after myocardial infarction [135, 136].

In summary, even if the picture appears to be quite complex, some of these changes are mediated by inflammatory pathways, such as NF-kB and AP-1, whereas the CREB pathway seems to be predominant in skeletal muscle [124]. On the other hand, overexpression of D2 in tanycytes, that has been observed in rats after LPS infusion [117, 137, 138], could be responsible for central suppression of the hypothalamic-pituitary-thyroid axis, thereby contributing to the complex picture of the regulation of thyroid function in this clinical condition.

## 5. Conclusion

**Figure 1:**
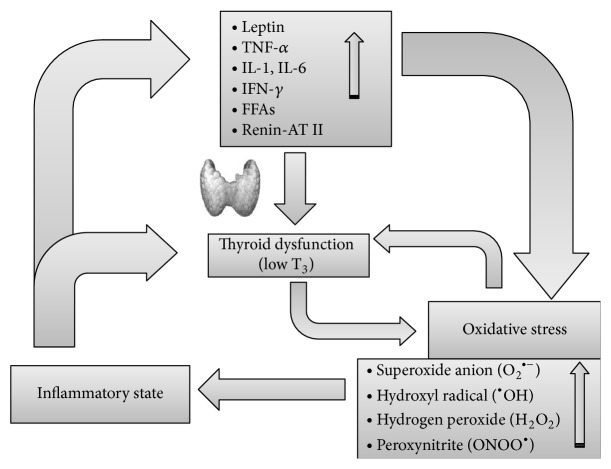
Proposed model of the interrelationships between inflammation, oxidative stress, and thyroid derangement. Inflammation, via hormone and cytokine changes, leads to oxidative stress and also affects thyroid function, causing nonthyroidal illness syndrome or pituitary-thyroid axis depression. At the tissue level, hypothyroidism reinforces the oxidative stress, which in turn worsens hypothyroidism by inhibiting deiodinases, thus establishing a vicious circle (see text for further explanations). AT: angiotensin; FFAs: free fatty acids; IFN: interferon; IL: interleukin; TNF: tumor necrosis factor.

**Figure 2:**
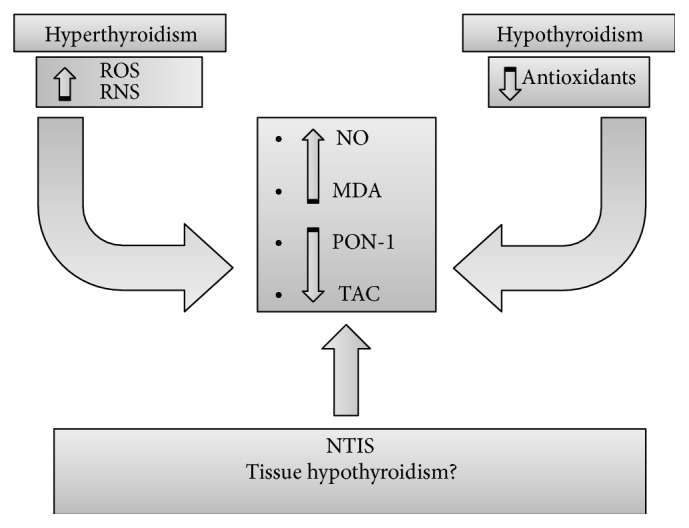
Both hyperthyroidism and hypothyroidism can cause oxidative stress but with different mechanisms. We speculate that nonthyroidal illness syndrome (NTIS) may represent a tissue hypothyroidism condition linked to intracellular and systemic oxidative stress. MDA: malondialdehyde; NO: nitric oxide; PON-1: paraoxonase-1; RNS: reactive nitrogen species; ROS: reactive oxygen species; TAC: total antioxidant capacity.

In conclusion, OS seems to be an important mechanism underlying the progress of inflammation. A vicious circle creates a link between these two conditions. Thyroid hormones can have a protective role, modulating antioxidant levels; on the other side, a tissue hypothyroidism can worsen OS (Figure 1). An interesting model is represented by NTIS, in which IL production due to inflammation can reduce the expression of deiodinases, inducing low-T_3_ levels and consequently a condition of tissue hypothyroidism. In turn, this latter could cause further OS (Figure 2). These pathophysiological observations suggest the possible therapeutic efficacy of antioxidants in the NTIS.
